# Hematological indices as predictors of in-hospital adverse outcomes in patients with COVID-19

**DOI:** 10.1590/1414-431X2026e15557

**Published:** 2026-10-12

**Authors:** E.B. Pordeus, N.A. Filgueira, C.R.P. Soares, P.S.R. de Araújo

**Affiliations:** 1 Programa de Pós-Graduação em Medicina Tropical, Universidade Federal de Pernambuco, Recife, PE, Brasil; 2 Hospital das Clínicas, Universidade Federal de Pernambuco/Empresa Brasileira de Serviços Hospitalares, Recife, PE, Brasil; 3 Centro de Ciências Médicas, Universidade Federal de Pernambuco, Recife, PE, Brasil; 4 Instituto Aggeu Magalhães, Fiocruz, Recife, PE, Brasil

**Keywords:** COVID-19, Neutrophil-lymphocyte ratio, Survival analysis, Hematological indices, Prognosis

## Abstract

The SARS-CoV-2 pandemic required medical teams to make rational decisions with limited resources, especially in Brazil. Various diagnostic tools, clinical guidelines, and existing indices were adopted to predict the risk of death and other adverse outcomes. The objective of this study was to validate three hematological indices [neutrophil-lymphocyte ratio (NLR), platelet-lymphocyte ratio (PLR), and lymphocyte-monocyte ratio (LMR)] for predicting adverse hospital outcomes in patients with COVID-19 in an observational cohort at a hospital in northeastern Brazil between April 2020 and October 2021. Hematological indices were calculated based on admission blood counts and correlated with hospital outcomes: in-hospital death, invasive mechanical ventilation, and renal replacement therapy. The analysis determined the frequencies of outcomes, established index thresholds, and assessed their association and accuracy and factors associated with hospital survival. Intra-hospital outcomes reflected disease severity; 34.62% of patients required admission to the ICU, 26.21% invasive mechanical ventilation, 4.2% renal replacement therapy, and 21.04% died. Advanced age, ICU requirement, and higher NLR were associated with higher risk of death. Multivariate analysis identified NLR as the only index associated with worse prognosis, with a cutoff point of 5.24 at hospital admission, showing a sensitivity of 84.0% and accuracy of 86.9%. The negative predictive value of 95.52% was highly significant, assisting hospital management by focusing efforts on higher-risk patients. These findings highlight the predictive value of the NLR, PLR, and LMR indices as valuable markers for risk stratification in patients hospitalized with COVID-19, offering a broader view of prognosis and guiding more personalized management and care strategies.

## Introduction

In Brazil, a total of 607,824 deaths and 21,810,855 confirmed infections were recorded from the beginning of the pandemic in March 2020 to October 2021, with mortality rates of 0.74 per 100,000 inhabitants (1). So far, Pernambuco has totaled 632,011 confirmed cases of the disease, with 54,571 severe cases (2). These figures have placed the country in an adversely prominent position on the global stage, characterizing it as second in terms of deaths worldwide (3).

The neutrophil-lymphocyte ratio (NLR), calculated by dividing the absolute number of neutrophils by the absolute number of lymphocytes, has been reported to have a significant value in indicating the patient's general inflammatory state, initially suggested in individuals with cancer facing situations of acute physical stress, such as sepsis or the postoperative period (4). After its increase, it was considered a risk marker for mortality, not only in malignancies (5), but also in acute coronary syndrome (6), intracerebral hemorrhage (7), polymyositis, and dermatomyositis (8).

At the beginning of the pandemic, research showed that severe cases of COVID-19 tended to have a higher NLR (9). It was questioned whether NLR, and other hematological indices such as platelet-lymphocyte ratio (PLR) and lymphocyte-monocyte ratio (LMR), could be considered independent predictors of mortality and what would be their cut-off points in hospitalized patients with COVID-19, as well as their applicability in Pernambuco, something that still needed to be elucidated.

Despite the presence of published meta-analyses (10) on these hematological indices, methodological differences and variability in cut-off points are evident, along with the underrepresentation of certain regions, such as South America. The aim of this study was to evaluate in patients infected with SARS-CoV-2 whether NLR, PLR, and LMR can predict adverse outcomes in patients hospitalized at the Federal University of Pernambuco Clinical Hospital.

## Material and Methods

### Study design

This was a retrospective cohort study based on the analysis of the medical records of patients (secondary data) admitted to the wards and intensive care units (ICU) of the UFPE Clinical Hospital with a confirmed diagnosis of COVID-19 through RT-PCR of nasal swab, between April 2020 and October 2021.

Pregnant women and patients with proliferative hematological diseases, undergoing chemotherapy for oncological diseases (solid or hematological tumors), physical trauma, immediate postoperative period, history of bleeding immediately prior to admission, and acute or chronic use of corticosteroids prior to admission were excluded. This exclusion is due to the possibility of altered results in the indices evaluated, making it difficult to predict adequately.

The outcomes studied were in-hospital death, need for invasive mechanical ventilatory assistance (IMVA) and renal replacement therapy, and in-hospital survival.

As independent variables, NLR, PLR, and LMR were studied, obtained from the admission blood count and categorized as elevated or not according to the cut-off point obtained in the ROC curve for each outcome.

### Ethical considerations

The study was developed in accordance with the Guidelines and Regulatory Standards for Research involving Human Beings (CNS/MS Resolution No. 466/2012). The Free and Informed Consent Form was waived because it was an observational, retrospective study. The registration and approval of the Ethics Committee of the UFPE Clinical Hospital/EBSERH can be accessed on the website: https://plataformabrasil.saude.gov.br/login.jsf with the number CAAE 60550322.1.0000.8807.

### Statistical analysis

Continuous variables are presented as median with interquartile range and means±SD and categorical variables as absolute frequencies and percentages.

Welch's *t*-test was used to compare means, and Pearson's chi-squared test was used to compare proportions.

To determine the cut-off points for NLR, PLR, and LMR and the outcomes of renal replacement therapy, invasive mechanical ventilation, and in-hospital death, ROC curves were constructed and the points with the best accuracy were selected.

To verify the association between NLR, PLR, LMR, and the outcomes of renal replacement therapy, invasive mechanical ventilation, and in-hospital death, crude and adjusted relative risks (RR) were calculated after multiple logistic regression and reported with a 95% confidence interval.

To evaluate the association between the NLR, PLR, LMR, and in-hospital survival, the log-rank test, Kaplan-Meier curves, and univariate and multivariate hazard ratios were determined by the Cox proportional regression method reported with a 95% confidence interval.

The R software package for Windows version 4.3.2 was used. P <0.05 was considered significant.

## Results

Between April 2020 and October 2021, the UFPE Clinical Hospital team hospitalized about 1166 patients with a flu-like syndrome. Among them, approximately 680 (58.31%) did not perform or did not test positive by RT-PCR for COVID-19.

Of the total, 486 (41.69%) patients tested positive for COVID-19 through the RT-PCR test. However, 177 (15.2%) individuals were excluded from the study: pregnant women, children, patients with a history of recent surgery, ongoing chemotherapy, transferred after more than 10 days of illness to another institution, and chronic use of corticosteroids. Thus, among the patients hospitalized at HC-UFPE with suspected COVID-19, 309 (26.5%) were considered eligible for the study (Figure 1).

**Figure 1:**
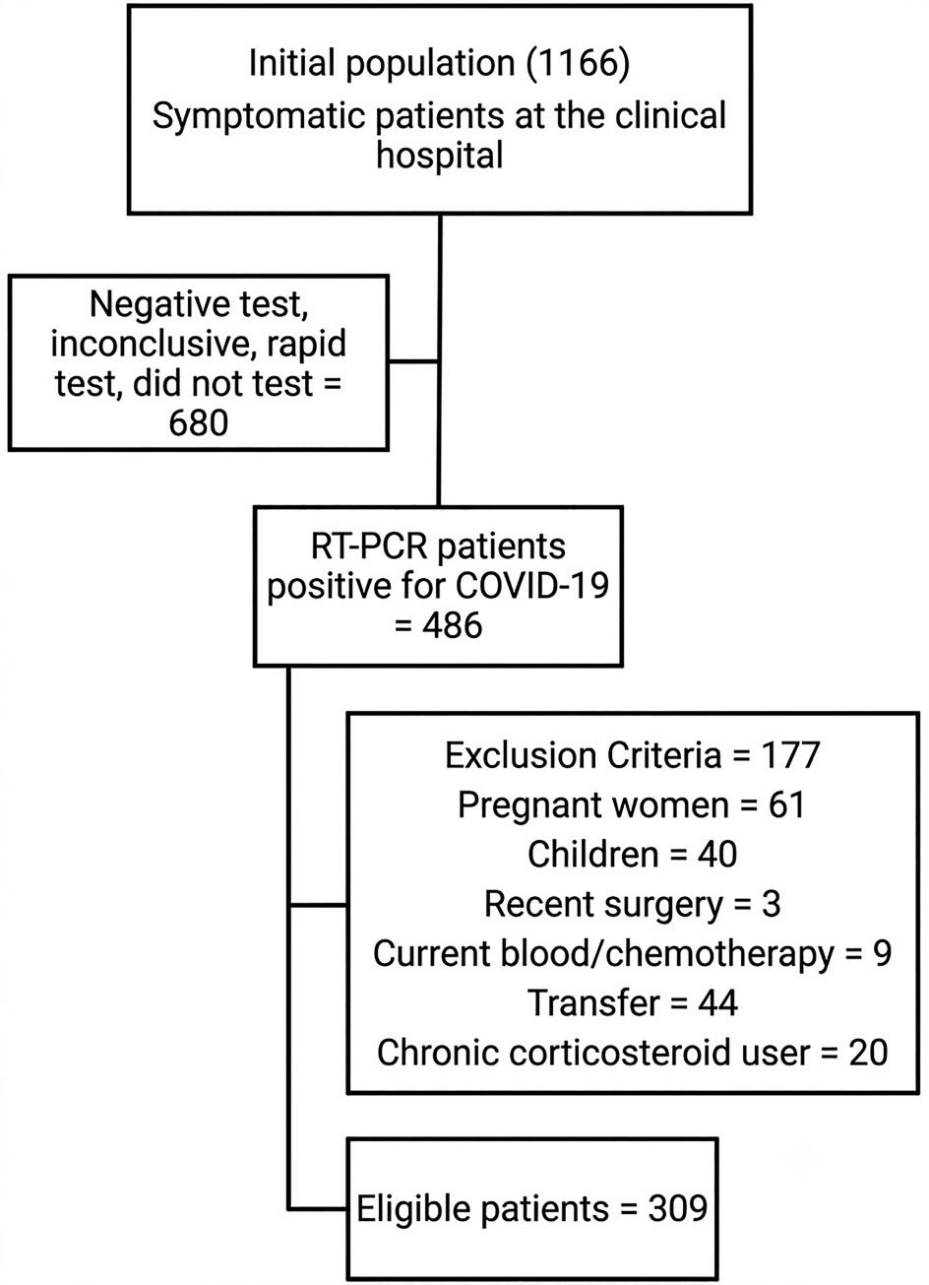
Flowchart of patient inclusion in the study.

The clinical and epidemiological characteristics of patients are shown in Table 1, with a mean age of 54.15 years and a standard deviation of 15.8 years. Of the total, 51.13% were male. Regarding comorbidities, 65.70% of patients had some pre-existing condition. The main comorbidities included systemic arterial hypertension (45.31%), diabetes mellitus (25.97%), and cardiovascular disease (14.56%). The least prevalent characteristics were rheumatologic disease (1.29%) and asthma (1.29%).

**Table 1:** Clinical, epidemiological, and laboratory characteristics of 309 patients hospitalized at HC-UFPE for COVID-19 between March 2020 and October 2021.

Characteristics	n (%)
Average age (years)	54.15±15.8
Male	158 (51.13%)
Comorbidities	203 (65.70%)
Systemic arterial hypertension	140 (45.31%)
Diabetes mellitus	80 (25.97%)
Cardiovascular disease	45 (14.56%)
Chronic kidney disease	38 (12.30%)
Obesity	31 (10.03%)
Tuberculosis	28 (9.06%)
Solid neoplasm	22 (7.12%)
Neurological disease	10 (3.24%)
PLHIV	7 (2.27%)
Chronic liver disease	6 (1.94%)
Major neurocognitive disorder	6 (1.94%)
Rheumatologic disease	4 (1.29%)
Asthma	4 (1.29%)
Hematological indices	
Medium NLR	3.58
Medium PLR	185.71
Medium LMR	2.06

PLHIV: person living with HIV/AIDS; NLR: neutrophil-lymphocyte ratio; PLR: platelet-lymphocyte ratio; LMR: lymphocyte-monocyte ratio. P<0.05, continuous variables compared using Welch's t-test; categorical variables compared using Pearson's chi-squared test.

Analyzing the target hematological indices of this study, the following medians were observed: NLR of 3.58, PLR of 185.71, and LMR of 2.06.

In-hospital outcomes reflected disease severity, where 107 patients (34.62%) required ICU care, while 81 (26.21%) required IMVA and 13 (4.2%) required renal replacement therapy (RRT), and 65 (21.04%) individuals died in the hospital (Table 2).

**Table 2:** In-hospital outcomes of 309 patients hospitalized at HC-UFPE for COVID-19 between March 2020 and October 2021.

Outcomes	n (%)
Median and IQR of length of hospital stay in days	6 (3-14)
Admission to an ICU unit	107 (34.62%)
Need for IMVA	81 (26.21%)
Need for RRT	13 (4.20%)
In-hospital death	65 (21.04%)

IQR: interquartile range; ICU: intensive care unit; IMVA: invasive mechanical ventilatory assistance; RRT: renal replacement therapy.

To determine the best cut-off points for the hematological indices NLR, PLR, and LMR in the prediction of in-hospital outcomes, ROC curves were constructed. For the NLR index, the cut-off points found were 5.24, 4.68, and 4.44. The areas under the curves (AUC) were 0.90, 0.84, and 0.71, respectively, associated with the outcomes of death, IMVA, and RRT. Regarding the PLR index, the cut-off points identified were 339.19, 185.47, and 185.47, with areas under the curves of 0.77, 0.77, and 0.62, also referring to the outcomes of death, IMVA, and RRT. Finally, for the LMR index, the optimal cut-off points were identified as lower than 1.50, 1.45, and 1.38, with areas under the curves of 0.84, 0.85, and 0.71, respectively, for death, IMVA and RRT (Figure 2 and Table 3).

**Figure 2:**
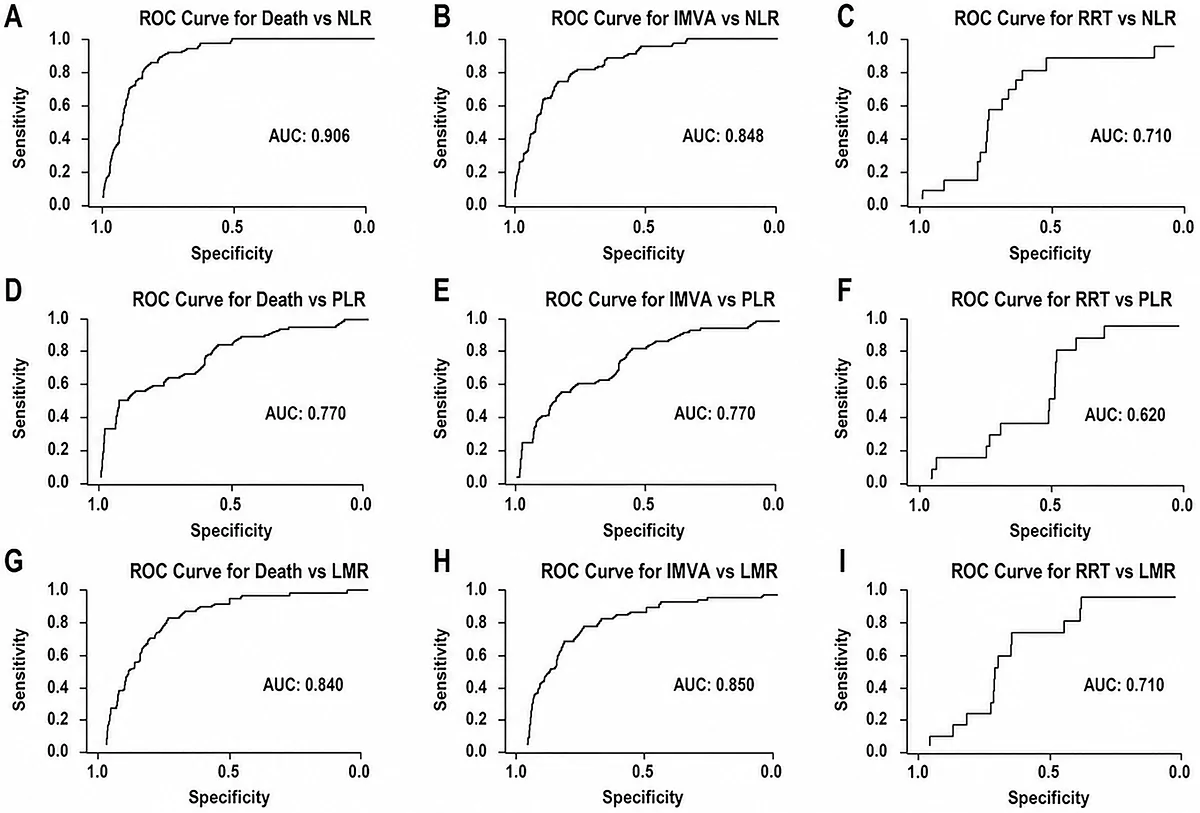
ROC curves for identification of neutrophil-lymphocyte ratio (NLR), platelet-lymphocyte ratio (PLR), and lymphocyte-monocyte ratio (LMR) cutoff points and in-hospital outcomes of 309 patients admitted to HC-UFPE for COVID-19 between March 2020 and October 2021: **A**, death according to NLR; **B**, IMVA according to NLR; **C**, RRT according to NLR; **C**, RRT according to NLR; **D**, death according to PLR; **E**, IMVA according to PLR; **F**, RRT according to PLR; **G**, death according to LMR; **H**, IMVA according to LMR; and **I**, RRT according to LMR. ROC: receiver operating characteristic; IMVA: invasive mechanical ventilatory assistance; RRT: renal replacement therapy; AUC: area under the curve. Cutoff points determined by Youden's index.

**Table 3:** Association between the NLR, PLR, and LMR indices and in-hospital outcomes of 309 patients hospitalized at HC-UFPE for COVID-19 between March 2020 and October 2021.

	Yes	No	RR (95%CI)	P
Indexes (cut-off points)	In-hospital death			
NLR				
≥5.24	57 (87.69%)	39 (15.98%)	3.62 (2.50-5.23)	0.001
<5.24	8 (12.31%)	205 (84.02%)	1	-
PLR				
≥339.19	35 (53.84%)	28 (11.47%)	2.20 (1.62-3.00)	0.001
<339.19	30 (46.16%)	216 (88.53%)	1	-
LMR				
≤1.50	53 (81.54%)	57 (23.36%)	7.60 (4.04-14.34)	0.001
>1.50	12 (18.46%)	187 (76.64%)	1	-
Indexes (cut-off points)	Renal replacement therapy			
NLR				
≥4.68	10 (76.93%)	104 (35.13%)	6.15 (1.61-23.49)	0.007
<4.68	3 (23.07%)	192 (64.87%)	1	-
PLR				
≥185.47	11 (84.61%)	145 (48.98%)	5.73 (1.50-37.43)	0.024
<185.47	2 (15.39%)	151 (51.02%)	1	-
LMR				
≤1.38	10 (76.93%)	92 (31.08%)	7.39 (1.99-27.49)	0.003
>1.38	3 (23.07%)	204 (68.92%)	1	-
Indexes (cut-off points)	Invasive mechanical ventilatory assistance			
NLR				
≥4.44	65 (80.24%)	56 (24.56%)	12.48 (6.68-23.30)	0.001
<4.44	16 (19.76%)	172 (75.44%)	1	-
PLR				
≥185.47	66 (81.48%)	90 (39.57%)	6.74 (3.62-12.54)	0.001
<185.47	15 (18.52%)	138 (61.43%)	1	-
LMR				
≤1.45	63 (77.77%)	43 (18.85%)	15.05 (8.08-27.99)	0.001
>1.45	18 (22.23%)	185 (81.15%)	1	-

NLR: neutrophil-lymphocyte ratio; PLR: platelet-lymphocyte ratio; LMR: lymphocyte-monocyte ratio; RR: relative risk; CI: confidence interval. Associations estimated by binary logistic regression.

Analyzing the association between the NLR, PLR, and LMR indices and the in-hospital outcomes, a higher risk of death, RRT, and IMVA was observed, with higher values of NLR and PLR and lower values of LMR (Table 3).

Regarding in-hospital death, patients with NLR above 5.24, PLR above 339.19, and LMR below 1.5 had a high risk of mortality, with relative risks of 3.62 (95%CI: 2.50-5.23), 2.20 (95%CI: 1.62-3.00), and 7.60 (95%CI: 4.04-14.34), respectively.

Similarly, patients with NLR above 4.68 (RR 6.15, 95%CI: 1.61-23.49), PLR above 185.47 (RR 5.73, 95%CI: 1.50-37.43), and LMR below 1.45 (RR 7.39, 95%CI: 1.99-27.49) were at higher risk of requiring RRT.

For IMVA, NLR values above 4.44, PLR above 185.47, and LMR less than 1.38 were associated with a significantly higher risk, with relative risks of 12.48 (95%CI: 6.68-23.30), 6.74 (95%CI: 3.62-12.54), and 15.05 (95%CI: 8.08-27.99), respectively.

The parameters for evaluating hematological indices in predicting hospital outcomes are shown in Table 4. Sensitivity values ranged from 53.8 to 84.01% and specificity values ranged from 51 to 88.5%. Negative predictive values ranged from 87.8 to 98.9%, while positive predictive values of the indices for predicting death and IMVA ranged from 48 to 64%, but for predicting RRT ranged from 7.1 to 9.8%. The highest positive likelihood ratio (LR+) value was 6.83 and the lowest negative likelihood ratio (LR-) value was 0.18, both referring to NLR in predicting death.

**Table 4:** Evaluation parameters of hematological indices for prediction of in-hospital outcomes in 309 patients hospitalized at HC-UFPE for COVID-19 between March 2020 and October 2021.

Diagnostic test endpoints	NLR			PLR			LMR		
	Death	IMVA	RRT	Death	IMVA	RRT	Death	IMVA	RRT
Cut-off point	5.24	4.68	4.44	339.19	185.47	185.47	1.50	1.45	1.38
AUC	0.906	0.848	0.710	0.776	0.774	0.624	0.844	0.857	0.716
Sensitivity	84.01%	78.50%	84.61%	53.84%	81.48%	84.61%	81.53%	77.77%	76.92%
Specificity	87.69%	80.24%	62.83%	88.52%	60.52%	51.01%	76.63%	81.14%	68.91%
PPV	64.71%	58.70%	09.10%	55.60%	42.30%	7.10%	48.20%	59.40%	9.80%
NPV	95.52%	91.50%	98.90%	87.80%	90.20%	98.60%	93.90%	91.11%	98.70%
LR+	6.83	3.98	2.28	4.69	2.07	1.73	3.49	3.81	2.72
LR-	0.18	0.27	0.24	0.52	0.31	0.30	0.24	0.20	0.22
Accuracy	86.90%	79.90%	63.80%	81.20%	66.00%	52.40%	77.70%	80.30%	69.20%

AUC: area under the curve; PPV: positive predictive value; NPV: negative predictive value; LR+: positive likelihood ratio; LR-: negative likelihood ratio; NLR: neutrophil-lymphocyte ratio; PLR: platelet-lymphocyte ratio; LMR: lymphocyte-monocyte ratio; IMVA: invasive mechanical ventilatory assistance; RRT: renal replacement therapy. Cutoff values determined by receiver operating characteristic (ROC) curve analysis using Youden's index.

Observing the factors possibly related to in-hospital survival (Table 5), the univariate analysis showed a significant association with age (HR 1.04; 95%CI: 1.02-1.05), systemic arterial hypertension (SAH) (HR 2.26; 95%CI: 1.33-3.84), cardiovascular disease (CVD) (HR 2.36; 95%CI: 1.35-4.11), solid cancer (HR 2.24; 95%CI: 1.18-4.25), need for ICU admission (HR 34.01; 95%CI: 12.35-93.69), NLR ≥5.24 (HR 14.47; 95%CI 6.88-30.4), PLR ≥339.19 (HR 14.47; 95%CI 6.88-30.4), and LMR ≤1.50 (HR 8.21; 95%CI: 4.3-15.4).

**Table 5:** Factors associated with in-hospital survival of 309 patients hospitalized at HC-UFPE for COVID-19 between March 2020 and October 2021.

Feature	HR (95%CI)	P	Adjusted HR (95%CI)	P
Age	1.04 (1.02-1.05)	<0.001	1.03 (1.01-1.05)	0.011
Sex	0.76 (0.47-1.25)	0.287		
Comorbidities	1.39 (0.70-2.78)	0.345		
SAH	2.26 (1.33-3.84)	0.002	0.78 (0.38-1.60)	0.495
DM	1.23 (0.73-2.05)	0.435		
CVD	2.36 (1.35-4.11)	0.003	0.75 (0.38-1.48)	0.404
Cirrhosis	0.85 (0.12-6.14)	0.870		
Asthma	0.00 (0.00-Inf)	0.996		
PLHIV	0.63 (0.09-4.59)	0.652		
Solid Cancer	2.24 (1.18-4.25)	0.013	0.87 (0.37-2.01)	0.738
TBG	1.53 (0.69-3.39)	0.293		
CKD	1.10 (0.56-2.18)	0.776		
Obesity	0.28 (0.07-1.16)	0.080		
Neurological disorders	0.24 (0.06-1.06)	0.060		
MND	0.41 (0.06-2.97)	0.377		
Rheumatological diseases	2.74 (0.98-7.69)	0.055		
ICU admission	34.01 (12.35-93.69)	<0.001	15.83 (5.26-47.68)	<0.001
Need for IMVA	0.58 (0.24-1.43)	0.239		
Need for RRT	1.84 (0.98-3.44)	0.057		
NLR ≥5.24	14.47 (6.88-30.4)	<0.001	4.08 (1.51-10.98)	0.005
PLR ≥339.19	6.01 (3.66-9.86)	<0.001	1.66 (0.81-3.44)	0.168
LMR ≤1.50	8.21 (4.37-15.4)	<0.001	0.99 (0.42-2.32)	0.982

SAH: systemic arterial hypertension; DM: diabetes mellitus; CVD: cardiovascular disease; PLHIV: person living with HIV/AIDS; HIV: human immunodeficiency virus; TBG: Smoking; CKD: chronic kidney disease; MND: major neurocognitive disorder; ICU: intensive care unit; IMVA: invasive mechanical ventilatory assistance; RRT: renal replacement therapy; NLR: neutrophil-lymphocyte ratio; PLR: platelet-lymphocyte ratio; LMR: lymphocyte-monocyte relationship. HR: hazard ratio; CI: confidence interval. Univariate and multivariate analyses performed using Cox proportional hazards regression model.

After multivariate analysis, the following variables were independently associated with a higher risk of in-hospital death: age (HR 1.03; 95%CI 1.01-1.05), need for ICU admission (HR 15.83; 95%CI: 5.26-47.68), and NLR greater than or equal to 5.24 (HR 4.08; 95%CI: 1.51-10.98) (Table 5).

Figure 3 shows the probability of in-hospital survival according to NLR cutoff point of 5.34 (P=0.017).

**Figure 3:**
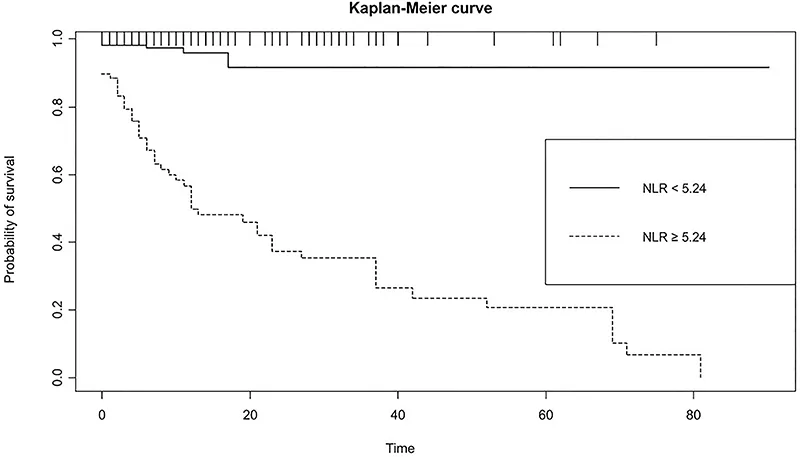
Kaplan-Meier curve for estimating in-hospital survival (in days) of 309 patients hospitalized at HC-UFPE for COVID-19 between March 2020 and October 2021 according to neutrophil-lymphocyte ratio (NLR) (P=0.017). Survival curves estimated by the Kaplan-Meier method. Groups compared using the log-rank test.

## Discussion

The results of this study showed that several hematological indices, when assessed at hospital admission, were associated with death in patients hospitalized for COVID-19 in a hospital in the northeast region of Brazil, with satisfactory accuracy to predict death in these patients, in addition to other outcomes such as RRT and the need for invasive mechanical ventilation (11).

In the present study, the frequency of death (21.04%) was lower than in the Brazilian review (38.0% in total and 48% of cases in the Northeast region). A possible explanation for this difference was the study period, since the previous study included only cases from the first months of 2020, when there was limited knowledge about the virus and inadequate infrastructure to implement support and prevention measures for the population, a common scenario at the beginning of the pandemic globally (12-14).

The body's first line of defense against external agents, such as bacteria and fungi, is innate immunity. The cellular components involved include macrophages, monocytes, dendritic cells, and, in particular, certain neutrophils and lymphocytes (such as natural killer cells) (13). Typically, in severe cases of COVID-19, a significant inflammatory response occurs due to the recruitment of neutrophils and their infiltration into various organs and tissues, leading to the formation of neutrophil extracellular traps (NETs) (14). This may be associated with acute lung injury, acute respiratory distress syndrome, septic shock, thrombosis, and dysfunction of multiple organs and systems (14), increasing the need for mechanical ventilation, intensive care unit admission, and leading to death.

Hospitalized COVID-19 patients who required intensive care generally exhibited predominantly elevated neutrophil counts, which were associated with a higher risk of death (15). The decrease in lymphocytes occurs through various mechanisms; early lymphocyte apoptosis is thought to result from pro-inflammatory cytokines, which influence the formation of B and T lymphocytes (16). Furthermore, activated monocytes from COVID-19 patients recruit and activate platelets, consistent with a dysregulated amplification loop that is associated with severity and mortality in COVID-19 patients (17).

Since the beginning of the COVID-19 pandemic, several studies have analyzed the association of NLR with disease outcomes. In previous studies, NLR, PLR, and LMR were studied as prognostic indexes in several clinical entities, including malignancies (5), acute coronary syndrome (6), intracerebral hemorrhage (7), polymyositis, and dermatomyositis (8).

In a meta-analysis, Alkhatib et al. (18) highlighted that NLR is a hematological index used to predict the severity of COVID-19, with a cutoff point above 3.63. They evaluated 8,120 patients and found strong diagnostic accuracy with an AUC of 0.88. In addition, in the meta-analysis by Ulloque-Badaracco et al. (19), in 2021, the analysis was expanded to 15,522 patients, examining NLR as an indicator of both severity and mortality. Despite variation in cutoff points and lack of consensus, increased severity and mortality were observed with each unit increase in NLR. In their meta-analysis of 26,098 patients in 2022, Sarkar et al. (10) also found that those with high NLR had worse outcomes. Although heterogeneity was a challenge, the usefulness of the NLR as a predictive tool was confirmed.

It is notable that these studies, despite methodological differences, converge on the importance of NLR. Variability in cutoff points reflects the diversity of the populations studied. The underrepresentation of certain regions, such as South America, highlights the need for more research that includes greater geographic variety.

During the first half of the pandemic in southern Brazil, the NLR cutoff value was 15.2, with a sensitivity of 83.3%, specificity of 86.2%, accuracy of 85.5%, NPV of 94.3%, and an AUC of 0.85 for predicting in-hospital death (20). In contrast, in northeastern Brazil, the values were lower, with sensitivity and specificity of 67% and 60%, respectively, associating an NLR greater than 10.6 with worse outcomes (21).

A study in Mato Grosso evaluated patients using the NLR and MLR indices (unlike our study, which evaluated the LMR), establishing cutoff values of 6.13 for the NLR and 0.73 for the MLR. The mortality rate was 17.1%, with sensitivity and specificity for the NLR of 78 and 67%, respectively, and for the MLR of 53 and 87%, respectively. Unlike the others, this study was conducted at two centers, one private and one public, in the central-western region of Brazil (22). None of the studies mentioned above performed a detailed survival analysis or an analysis of validity parameters as ours did. Furthermore, there are no studies in Pernambuco to date that have evaluated these hematological indices as this study has.

The NLR was found to be elevated in patients hospitalized for COVID-19 and was significantly higher in those who died. With a cutoff point of 5.24 at hospital admission, the sensitivity and accuracy of the NLR in identifying patients at higher risk of death were satisfactory: 84.0 and 86.9%, respectively. The negative predictive value (NPV) is particularly relevant in the HC-UFPE study, especially for identifying patients at lower risk of developing severe complications. For example, for the NLR index, the NPV was 95.52% for death, indicating that patients with values below the cut-off point have a very low probability of death. This finding is useful for guiding patients and families and optimizing hospital management, focusing efforts on patients at higher risk (22).

The absence of similar studies in Pernambuco to date, along with the inclusion of survival analyses and detailed parameter validation, makes this study useful for clinical practice and research in hematology and infectious diseases. The high NPV found for NLR, in particular, stands out as a crucial finding, providing a solid foundation for potential guidelines on screening and management of COVID-19 patients in hospital settings.

Regarding the factors associated with in-hospital survival, univariate analysis showed an association with age, need for intensive care, some comorbidities (hypertension and CVD), and the hematological indices studied. After multivariate analysis, age, need for ICU, and NLR remained independently associated ([Bibr B22] [Bibr B23] 2224).

A relationship was observed between mortality and individuals requiring ICU admission, but the multivariate analysis could not identify the true impact of these comorbidities on increased COVID-19 mortality. Various studies have addressed the impact of COVID-19 in the ICU, highlighting issues ranging from bed occupancy to challenges faced by health systems. Pereira (25) discussed the pandemic's trajectory in a university hospital in Porto Alegre during critical times related to ICU bed occupancy and social isolation measures adopted. Another issue was the distribution and availability of ICU beds and mechanical ventilators in Brazil, identifying a significant shortage of these resources in several regions, mainly in the Northeast, and linked this to higher mortality rates associated with COVID-19 (26).

The present study adds valuable information to the literature about the ability of NLR to predict unfavorable in-hospital outcomes in patients with COVID-19, presenting cut-off points for each outcome and a complete analysis of the validity parameters of these tests. In addition, a population from the Northeast using the public health service, underrepresented in previous studies, was studied.

On the other hand, the study had some limitations. We determined that hematological indices would be calculated based on the blood count at hospital admission. However, the admission date to HC-UFPE varied greatly in relation to the progression of the disease, which may have introduced bias in the study. In the early months of the pandemic, due to overcrowding in referral services, patients often spent many days in emergency departments waiting for an intensive care vacancy. Another limitation was selecting patients from only one university center over a varied period of time, which may have introduced biases due to the influence of vaccination effects, therapeutic options, treatment expertise during the pandemic, and lack of evaluation of COVID-19 variants.

Despite this, the results confirm observations by other researchers and suggest that these hematological parameters may be useful in clinical practice to assess the prognosis of patients hospitalized for COVID-19 in relation to the risk of death and the need for RRT and IMVA.

These findings show that, in addition to their predictive value, the NLR, PLR, and LMR indices are valuable markers for risk stratification in patients hospitalized for COVID-19, providing a more comprehensive overview of the prognosis of these patients and the need for more personalized management and care strategies for these patients.

## Data Availability

The data that support the findings of this study are available from the corresponding author upon reasonable request.
